# The TROG 15.01 stereotactic prostate adaptive radiotherapy utilizing kilovoltage intrafraction monitoring (SPARK) clinical trial database

**DOI:** 10.1002/mp.17529

**Published:** 2024-11-23

**Authors:** Chandrima Sengupta, Doan Trang Nguyen, Yifan Li, Emily Hewson, Helen Ball, Ricky O'Brien, Jeremy Booth, John Kipritidis, Thomas Eade, Andrew Kneebone, George Hruby, Regina Bromley, Peter Greer, Jarad Martin, Perry Hunter, Lee Wilton, Trevor Moodie, Amy Hayden, Sandra Turner, Nicholas Hardcastle, Shankar Siva, Keen‐Hun Tai, Sankar Arumugam, Mark Sidhom, Per Poulsen, Val Gebski, Alisha Moore, Paul Keall

**Affiliations:** ^1^ Image X Institute The University of Syndey Sydney Australia; ^2^ School of Health and Biomedical Sciences RMIT University Melbourne Australia; ^3^ Norther Sydney Cancer Centre Royal North Shore Hospital Sydney Australia; ^4^ Department of Radiation Oncology Calvary Mater Newcastle Newcastle Australia; ^5^ Crown Princess Mary Cancer Centre Westmead Hospital Sydney Australia; ^6^ Department of Physical Sciences Peter MacCallum Cancer Centre Melbourne Australia; ^7^ Liverpool and Macarthur Cancer Therapy Centres Liverpool Hospital Liverpool Australia; ^8^ Department of Oncology Aarhus University Hospital and Danish Centre for Particle Therapy Aarhus University Hospital Aarhus Denmark; ^9^ NHMRC Clinical Trials Centre University of Sydney Sydney Australia; ^10^ TROG Cancer Research Waratah Australia

**Keywords:** Intrafraction tumor motion, Prostate SABR dataset, Real‐time 6DoF prostate motion

## Abstract

**Purpose:**

The US National Institutes of Health state that *Sharing of clinical trial data has great potential to accelerate scientific progress and ultimately improve public health by generating better evidence on the safety and effectiveness of therapies for patients* (https://www.ncbi.nlm.nih.gov/books/NBK285999/ accessed 2024‐01‐24.). Aligned with this initiative, the Trial Management Committee of the Trans‐Tasman Radiation Oncology Group (TROG) 15.01 Stereotactic Prostate Adaptive Radiotherapy utilizing Kilovoltage intrafraction monitoring (KIM) (SPARK) clinical trial supported the public sharing of the clinical trial data.

**Acquisition and Validation Methods:**

The data originate from the TROG 15.01 SPARK clinical trial. The SPARK trial was a phase II prospective multi‐institutional clinical trial (NCT02397317). The aim of the SPARK clinical trial was to measure the geometric and dosimetric cancer targeting accuracy achieved with a real‐time image‐guided radiotherapy technology named KIM for 48 prostate cancer patients treated in 5 treatment sessions. During treatment, real‐time tumor translational and rotational motion were determined from x‐ray images using the KIM technology. A dose reconstruction method was used to evaluate the dose delivered to the target and organs‐at‐risk. Patient‐reported outcomes and toxicity data were monitored up to 2 years after the completion of the treatment.

**Data Format and Usage Notes:**

The dataset contains planning CT images, treatment plans, structure sets, planned and motion‐included dose‐volume histograms, intrafraction kilovoltage, and megavoltage projection images, tumor translational and rotational motion determined by KIM, tumor motion ground truth data, the linear accelerator trajectory traces, and patient treatment outcomes. The dataset is publicly hosted by the University of Sydney eScholarship Repository at https://doi.org/10.25910/qg5d‐6058.

**Potential Applications:**

The 3.6 Tb dataset, with approximately 1 million patient images, could be used for a variety of applications, including the development of real‐time image‐guided methods, adaptation strategies, tumor, and normal tissue control modeling, and prostate‐specific antigen kinetics.

## 1 INTRODUCTION

Prostate motion during cancer radiation therapy may shift the tumor outside the beam, simultaneously reducing target dose^1^ and exposing normal tissues to potentially damaging radiation doses.^1^ Targeted radiotherapy images the cancerous tumor in real‐time, enabling the treatment beam to focus its destructive energy on the patient's cancer, not their healthy tissues. Targeted radiotherapy on MRI‐Linacs, has halved side effects in prostate cancer patients.^2^ However, >95% of radiotherapy is given on conventional x‐ray guided linacs. This clinical benefit has led to a global demand: over 70% of centers want better targeted radiotherapy but are limited by resources and capacity.^3^

To enable accessible targeted radiotherapy, the real‐time tracking capabilities of MRI‐Linac solutions need to be adapted to x‐ray guided linacs. Many real‐time image‐guided radiation therapy (IGRT) approaches have been developed for x‐ray guided linacs. One of these technologies, kilovoltage intrafraction monitoring (KIM) was developed and investigated in the TROG 15.01 Stereotactic Prostate Adaptive Radiotherapy utilizing KIM (SPARK) clinical trial (NCT02397317).^1^ This manuscript presents the details of the SPARK clinical trial data acquired with the KIM technology on conventional radiation therapy systems.

The SPARK trial itself achieved a number of milestones, including the first quantification of the accuracy of in‐patient 6‐degree of freedom target tracking, the first multi‐center, multi‐vendor use of KIM, and the first clinical use of KIM with multi‐leaf collimator (MLC) tracking, demonstrating real‐time adaptive radiation therapy on a conventional x‐ray guided linac.^1^, ^4^, ^5^ The technological focus of the trial meant that a large amount of patient kilovoltage and megavoltage images were acquired along with planning and patient outcome data that could be of use to the community.

Aligned with the US National Institutes of Health initiative,† and the FAIR Principles,‡ the Trial Management Committee of the TROG 15.01 SPARK clinical trial supported the public sharing of the clinical trial data.

## 2 ACQUISITION AND VALIDATION METHODS

### 2.1 Overview of dataset

The data originate from the TROG 15.01 SPARK clinical trial.^1^, ^6^ The SPARK trial was a phase II prospective multi‐institutional clinical trial approved by the Hunter New England Local Health District Human Research Ethics Committee, (HREC/15/HNE/216) and registered with clinicaltrials.gov (NCT02397317). The inclusion criteria were patients aged 18 years or older, histologically proven prostate adenocarcinoma, low or intermediate risk disease as defined by the NCCN guidelines: (a) Low Risk: All of prostate‐specific antigen (PSA) < 10 ng/mL, Gleason Grade 6 AND Stage T1 or T2a (b) Intermediate Risk: Any or all of PSA 10–20 ng/mL, Gleason Grade 7 OR Stage T2b‐c (c) Absence of high‐risk features (PSA > 20, T3‐4, N1 or M1 disease, Gleason score 8–10) with PSA measured within 3 months prior to enrolment, ECOG Performance status 0–2, suitable for definitive external beam radiotherapy (intensity modulated radiation therapy [IMRT] or volumetric modulated arc therapy [VMAT]), ability to have three gold fiducial markers placed in the prostate.

The aim of the SPARK clinical trial was to measure the geometric and dosimetric cancer targeting accuracy achieved with the KIM technology for 48 prostate cancer patients treated in 5 treatment sessions. Patients received 7.25 Gy in a treatment session. A 5 mm clinical target volume (CTV) to planning target volume (PTV) margin was applied in all directions except the posterior direction, where a 3 mm expansion was used. VMAT planning was used, and both flattened and flattening filter‐free beams were used.

The immobilization and patient‐specific quality assurance varied between the five trial sites. All patients were treated in the supine position, Immobilization devices included head support, knee blocks or pillows, and feet locks. Patient‐specific quality assurance included treatment plan dose measurements using either volumetric detectors or portal dosimetry as well as independent dose calculation validation.

The clinical trial was performed at five radiotherapy centers situated in New South Wales and Victoria in Australia. During treatment, real‐time tumor translational and rotational motion were determined from x‐ray images using the KIM technology. Two methods were implemented to adapt to tumor motion during treatment: (1) gating by applying KIM‐triggered couch shifts and (2) MLC tracking. A dose reconstruction method was used to evaluate the dose delivered to the target and organs‐at‐risk with and without real‐time KIM monitoring.^7^ Patient‐reported outcomes and toxicity data were collected up to 2 years after the completion of the treatment.

### 2.2 Data acquisition

**FIGURE 1:**
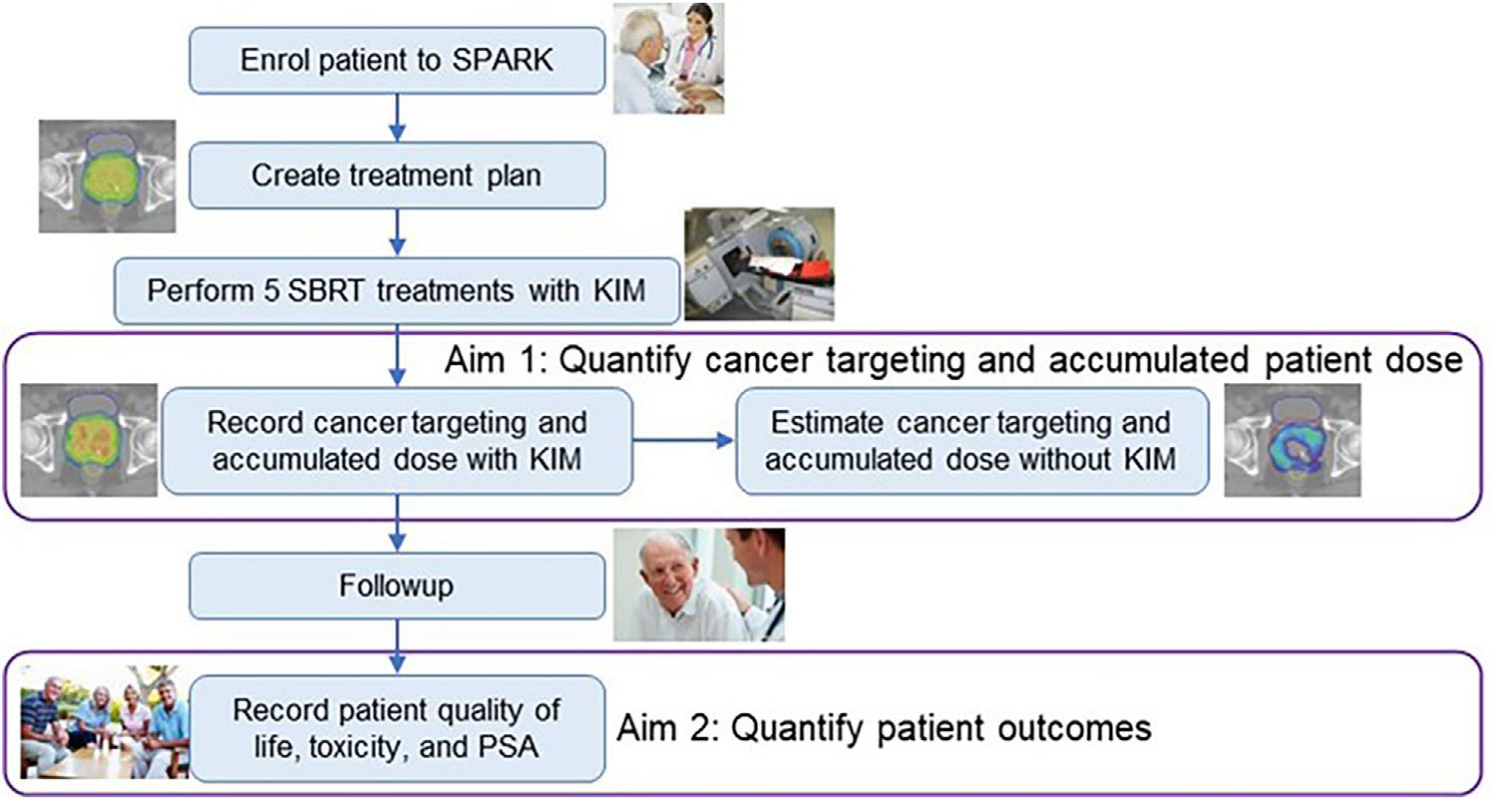
The SPARK trial patient journey and outcome analyses. Adapted from the published SPARK protocol paper.^6^

The SPARK trial patient journey and outcome analyses are shown in Figure 1. The KIM technology used gold fiducial markers (3–5 mm in length) as a surrogate for the prostate tumor motion which were implanted near the tumor in accordance with the standard protocol at each participating institution. The KIM technology used the on board kilovoltage x‐ray imager and segmented each fiducial marker based on a template matching method. The KIM method converted 2D positions from the kilovoltage x‐ray system acquired at 10 Hz (on Varian) and 5.5 Hz (on Elekta) into a real‐time three‐dimensional (3D) position of the tumor during radiotherapy treatment. The 3D marker position was reconstructed by use of a 3D Gaussian probability density function (PDF), which was estimated prior to treatment from the setup CBCT projections.^4^ During treatment, the PDF was updated with the new‐incoming fluoro kilovoltage images. The centroid position of the prostate was determined by the average of the three marker positions. The rotational motion was calculated from the 3D marker position using an iterative closest point algorithm.^8^

The SPARK data were collected from five radiotherapy centers. CT images, structure sets, dose DICOMs, and treatment plans are available in DICOM format. For real‐time intrafraction imaging and tracking, KIM used HIS, TIFF, and HND image formats, as writing full DICOM images in real‐time is slower. For each center, a brief description of the intrafraction kilovoltage and Megavoltage image collection method is given in Table 1. The SPARK study guidelines recommended, but did not require, a KIM gating tolerance to pause the beam if target motion > 2 mm from isocenter in any axis persisted for > 5 s. When the motion exceeded this tolerance, a KIM‐triggered couch shift was performed to compensate for this motion. Centers 1, 2, and 5 followed this recommendation. At center 3, the KIM gating criteria was set for any motion > 3 mm persisting for > 5 s in any axis. Center 4 performed real‐time MLC tracking for motion adaptation.^4^, ^5^

**TABLE 1:** The SPARK intrafraction kilovoltage and megavoltage image data collection method for the five participating centers.

Site	No. of patients	Linear accelerator	Motion correction (KIM gating criteria)	Image type	Projection angle source	Projection angle type
Center 1	9	Varian TrueBeam	Gating (2 mm/5 s)	.tiff	The last number in the image name is separated by “_*”*.	MV gantry
Center 2	20	Varian TrueBeam	Gating (2 mm/5 s)	.tiff	The last number in the image name is separated by “_*”*.	MV gantry
Center 3	5	Varian TrueBeam	Gating (3 mm/ 5 s)	.tiff	The last number in the image name is separated by “_*”*.	MV gantry
Center 4	10	Varian Trilogy	MLC tracking	.hnd	Image header	MV gantry
Center 5	4	Elekta	Gating (2 mm/5 s)	.his	Frames.xml	MV gantry

### 2.3 VALIDATION METHODS

Validation methods consisted of pre‐ and post‐treatment quality assurance of treatment plans and delivery, KIM‐specific quality assurance processes,^9^ and post‐treatment patient measured comparison of the KIM results to kilovoltage/megavoltage triangulation results,^10^ as expanded upon below.

#### 2.3.1 Validation of treatment plans and delivery

TROG was responsible for the SPARK trial radiotherapy quality assurance. This included comprehensive pre‐treatment and post‐treatment centralized review of radiotherapy data.

All trial participants underwent centralized individual case review of the radiotherapy treatment plan (dose, technique, contours) and delivery. The radiotherapy treatment plan in DICOM format was submitted via the TROG Central Quality Management System (CQMS), at least seven days prior to the trial participant commencing treatment. Independent reviewers assessed the treatment plan through the use of a 3D imaging review program, MIM Maestro software. The SPARK independent review team comprised of six Radiation Oncologists, four Radiation Oncology Medical Physicists, and four Radiation Therapists.

48 participants underwent pre‐treatment review, from five radiotherapy centers, for which the review sampling was divided into two stages:

Stage 1: The first four participants from each center underwent full review of technical/dosimetric variables as well as a contour review.

Stage 2: One in four participants subsequently underwent full review (technical/dosimetric and contours). The remaining three in four participants from each center underwent technical/dosimetric review only. Sampling was performed randomly.

Dosimetric, technical, and contouring criteria defined in the protocol^6^ were classified according to established risk definitions (Table 2). Individual case feedback was provided prior to radiotherapy treatment commencement and included commentary from the reviewer/s, protocol variations, missing/in‐evaluable data, and overall review outcome of pass or resubmission. Four cases were resubmitted. Upon resubmission, all cases were deemed a pass.

**TABLE 2:** Individual case review classifications/definitions.

Criteria category	Description
Minor/Lesser variation	Variation that will not have a significant impact on the outcome or interpretation of the study but may require follow‐up or education to prevent recurrence in subsequent cases or progression to major deviations.
Major variation	Variation from protocol‐specified procedures that makes the resulting data questionable and may affect the interpretation of the endpoints.
Missing/In evaluable	Review is not possible based on the documentation provided.

A high level of protocol compliance was observed, at final review, only 4/48 (8.3%) cases retained one major variation (PTV D_max_ located within a critical structure, *n* = 2; intermediate dose spillage, *n* = 1; PTV volume > 90 cm^3^, *n* = 1). No replanning was performed using the KIM motion data.

#### 2.3.2 Validation of the KIM system

The KIM method was implemented at each treatment center using three KIM‐specific quality assurance tests including: (1) static localization accuracy, (2) dynamic localization accuracy, and (3) treatment interruption accuracy following Ng et al.^9^ Tests (1)–(3) were performed using KIM to measure static and representative patient‐derived prostate motion trajectories using a 3D programmable motion stage supporting an anthropomorphic phantom with implanted gold markers to represent the clinical treatment scenario. The mean and standard deviation for all three tests were <1 mm at all centers.

Post‐treatment, the geometric accuracy and precision of KIM were assessed from over 33 000 patient images (translation) and over 9000 images (rotation) by comparing the real‐time measured motion to retrospective kilovoltage/megavoltage triangulation.^10^

## 3 DATA FORMAT AND USAGE

The dataset contains 48 prostate cancer patient data including:

1. Treatment plans, planning CT images, RT structure sets, planned dose DICOMs, and planned marker positions (centroid files),
2. Intrafraction kilovoltage and megavoltage projection images acquired during treatment, translational and rotational motion of the tumor determined by KIM during the treatment sessions (KIM logs) and tumor motion ground truth data determined by the triangulation method,
3. The linear accelerator log files,
4. Motion‐included dose‐volume histograms calculated based on KIM‐measured motion and the ones calculated without KIM (simulated), and
5. Patient treatment outcomes.

**FIGURE 2:**
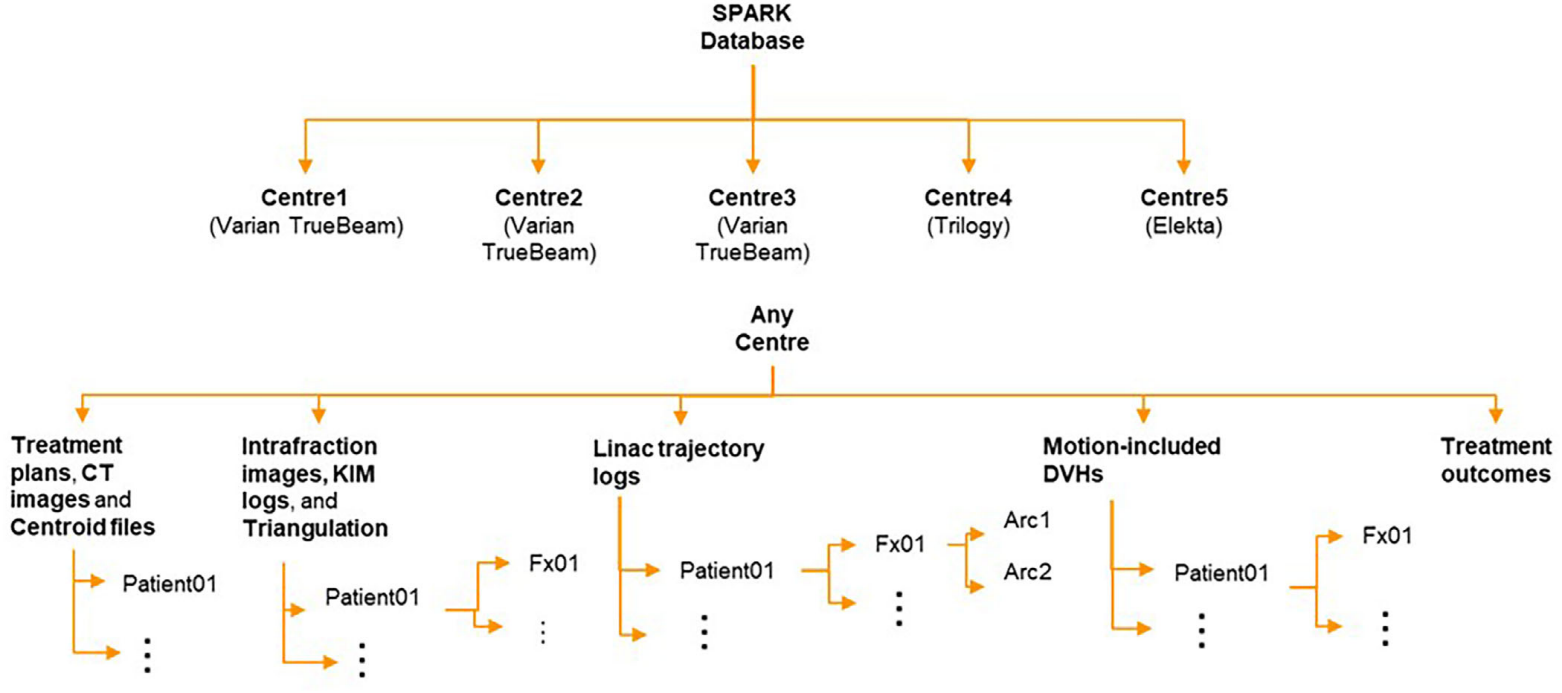
The SPARK database structure.

The dataset is publicly hosted by the University of Sydney eScholarship Repository at https://doi.org/10.25910/qg5d‐6058. The database structure is shown in Figure 2. Due to the large data size for centers 2 and 3, the data are split into smaller patient groups and stored in separate zipped folders.

Within each center, nine sub‐folders can be found, three of them defined at the patient level as listed below. A detailed description of each folder is:

**Folder 1 – Treatment plans**: Contains the treatment plans (RP), planned dose (RD), and structure sets (RS) in DICOM format.

**Folder 2 – CT images**: Contains simulation CT images in DICOM format.

**Folder 3 – Centroid files**: Contains the planned marker positions with respect to the treatment isocenter in the plain text file format.

The six other folders are defined at the fraction level as listed below.

**Folder 4 – Intrafraction images**: Each fraction contains two sub folders called “KV” and “MV” containing kilovoltage and MV intrafraction images acquired during every treatment fraction at 10 Hz (on Varian) and 5.5 Hz (on Elekta). An in‐house developed software^6^ was used to collect these images for Varian linear accelerators, whereas, for Elekta linear accelerators, the x‐ray volumetric imaging (XVI) system was employed to collect the kilovoltage images. megavoltage images were not available for the Elekta site. When the KIM software crashed during a treatment fraction due to technical problems, a new image folder was created as the KIM software had to be restarted. In this scenario, multiple subfolders exist named “Images1,*”* “Images2*”*… representing images for each KIM session during a treatment fraction.

**Folder 5 – KIM logs**: KIM‐derived translational motion and rotational motion of each marker were determined by analyzing the patient kilovoltage images. Each folder also contains a figure demonstrating the gantry rotation as a function of KIM‐measured time and indicates the datapoints belonging to the setup CBCT, as well as the datapoints that make up the rest of the treatment. KIM generates motion files named “*MarkerLocationsGA_Couchshift_x.txt”* (where, x = 0, 1, 2), where “*MarkerLocationsGA_Couchshift_0.txt”* denotes the first KIM log file with motion information written in the IEC coordinate system. Afterwards, every time, a couch shift is performed another “*MarkerLocationsGA_Couchshift_x.txt”* is created. Couch shift values are logged into “*Couchshifts.txt”* file written in the Varian IEC couch coordinate system for Varian systems and in IEC 1217 coordinate system for the Elekta system. The rotation of the marker centroid is written in the “*Rotation.txt”* file. The *MarkerLocationsGA_Couchshift_x.txt* file contains the following information:

*Time* (s): KIM starts counting time whenever the KIM software is started.

*Gantry*: kilovoltage gantry angle in degree.

*Marker_x_AP, Marker_x_LR*, *Marker_x_SI*: 3D position in mm of the Marker x in the anterior‐posterior direction, left‐right, superior–inferior direction, respectively in the patient coordinate system.

*Marker_x_X*: 2D segmented X position of the marker x in the kilovoltage imager plane.

*Marker_x_Y*: 2D segmented Y position of the marker x in the kilovoltage imager plane.

where, “x*”* denotes: Marker 0/1/2.

The next few columns denote the marker cross‐correlation coefficients with the last column denoting the image name for which the marker position was determined.

The KIM log analysis codes are available here: github.com/Image‐X‐Institute/SPARK_Data_Analysis_Code/tree/main/Analysis%20code.

**Folder 6 – Triangulation**: Contains the ground truth marker positions given in the “*TriangulatedPos.xls.”* Triangulation was performed using the analysis codes available here: *github.com/Image‐X‐Institute/SPARK_Data_Analysis_Code/tree/main/Analysis%20code/SPARK%20Triangulation%20Code*. Triangulation results are not available for the Elekta site.

**Folder 7 – Linac trajectory logs**: For Centers 1–3, this contains the linear accelerator trajectories in the .bin format acquired during a treatment fraction (See Varian Medical Systems Truebeam Trajectory Log File Specification—Document ID P1012906‐004‐D). For Center 4, the linear accelerator trajectories were recorded with the .dlg format (see Varian Medical Systems Dynalog File Viewer Reference Guide Version 8.X Revision 06). Several Dynalog files were collected during treatment and the files that correspond to the treatment arcs can be determined by the columns describing gantry rotation and beam on state. These files are not available for the Elekta site.

**Folder 8 – Motion‐included dose‐volume histograms (DVHs)**: This folder contains motion‐included DVHs. The motion‐included dose reconstruction was performed using the method described in *Poulsen et al*.^7^ There are two DVHs in each fraction; dose delivered with KIM guidance named “Pat_Fx_withKIM.txt*”* and simulated delivered dose without KIM guidance named “Pat_Fx_woKIM.txt.*”* In fractions with no KIM‐triggered gating events, there is only one DVH in the folder named “Pat_Fx_withKIM.txt.*”* For each patient, there are three DVHs at the patient level: “Planned_DVH.txt,*”* “Patx_PlanSumwithKIM.txt*”* representing the cumulative dose over five fractions with KIM guidance, and “Patx_PlanSumwoKIM.txt*”* representing simulated cumulative delivered dose without KIM guidance.

**Folder 9 – Treatment outcomes**: Patient outcomes were recorded at the timepoints of pretreatment (E1), during treatment (E2), 2‐week follow‐up (E3), 6‐week follow‐up (E4), 3‐month follow‐up (E5), 6‐month follow‐up (E6), 12‐month follow‐up (E7), 18‐month follow‐up (E8), 24‐month follow‐up (E9), 30‐month follow‐up (E10), 36‐month follow‐up (E11), 42‐month follow‐up (E12) and 48‐month follow‐up (E13). Treatment (E2) occurred over several weeks and there are variables representing each week (e.g., WeekFourProctitisAeGrade_E2) although the patients were not assessed every week. Empty cells indicate missing data (particularly at later timepoints) or data not requested for conditional variables.

a) **Patient adverse events**

The events were classified and graded at timepoints E1–E13 according to the Common Terminology Criteria for Adverse Events (CTCAE) version 4 (website link given below). This was the current version at the time the protocol was developed. https://ctep.cancer.gov/protocoldevelopment/electronic_applications/ctc.htm

The adverse events assessed were relevant to radiation treatment of prostate cancer with the addition of a field to enter other adverse events not listed in the CRF.

**Conditional Variables**

If an adverse event was graded as 1 or greater (or text was added for the Other Adverse Event variable), data for two additional variables were recorded.

Treatment Related—Scale of 0–5 with 0 being adverse event not related to treatment and 5 being definitely related to treatment.

Serious Adverse Event—No serious adverse events were recorded during the study.

b) **Patient PSA levels and disease progression**

PSA levels in ng/mL were measured at the timepoints E1, E4, E5, E6), E7, E8, E9, E10, E11, E12 (42‐month follow‐up) and E13 (48‐month follow‐up). These variables are named PSALevel_E*_C** where *is the timepoint and ** is the CRF number.

**Other variables**

**Biochemical Failure** (BCFDiagnosed; 0 = no BCF, 1 = BCF) according to the ASTRO Consensus Committee definition for BCF following primary treatment with radiotherapy as an increase in PSA by greater than or equal to 2 ng/mL above the treatment nadir.

**Cancer Identified** (prostate) (CancerIdentified; 0 = no, 1 = yes)

Conditional (depending on answers to BCFDiagnosed and CancerIdentified) variables are listed below.

If Yes (1) for **BCFDiagnosed** the follow‐up variables include:

**PSA Relapse** (PSARelapse; 0 = no, 1 = yes)

**Local Failure** (LocalFailure; 0 = no, 1 = yes)

**Distant Failure** (DistantFailure; 0 = no, 1 = yes)

**New Lesions Identified** (NewLesionsIdentified; 0 = no, 1 = yes)

**Bone Metastases Identified** (BoneMetastasesIdentified; 0 = no, 1 = yes)

**Androgen Deprivation Therapy Initiated** (ADTInitiated; 0 = no, 1 = yes)

If yes (1) for **Cancer Identified**, the follow‐up variable was:

Extent of disease using the criteria defined at https://www.cancer.gov/about‐cancer/diagnosis‐staging/staging.

c) **Patient‐reported outcomes**

Patient‐reported outcomes were collected with the EPIC‐26^11^ survey at the timepoints E1, E3, E4, E7, and E11. Where a survey was not completed or partially completed the cells are left empty. All the patients were surveyed up to 2 years timepoint. The raw survey responses were recorded.

### 3.1 Data anonymization software

**FIGURE 3:**
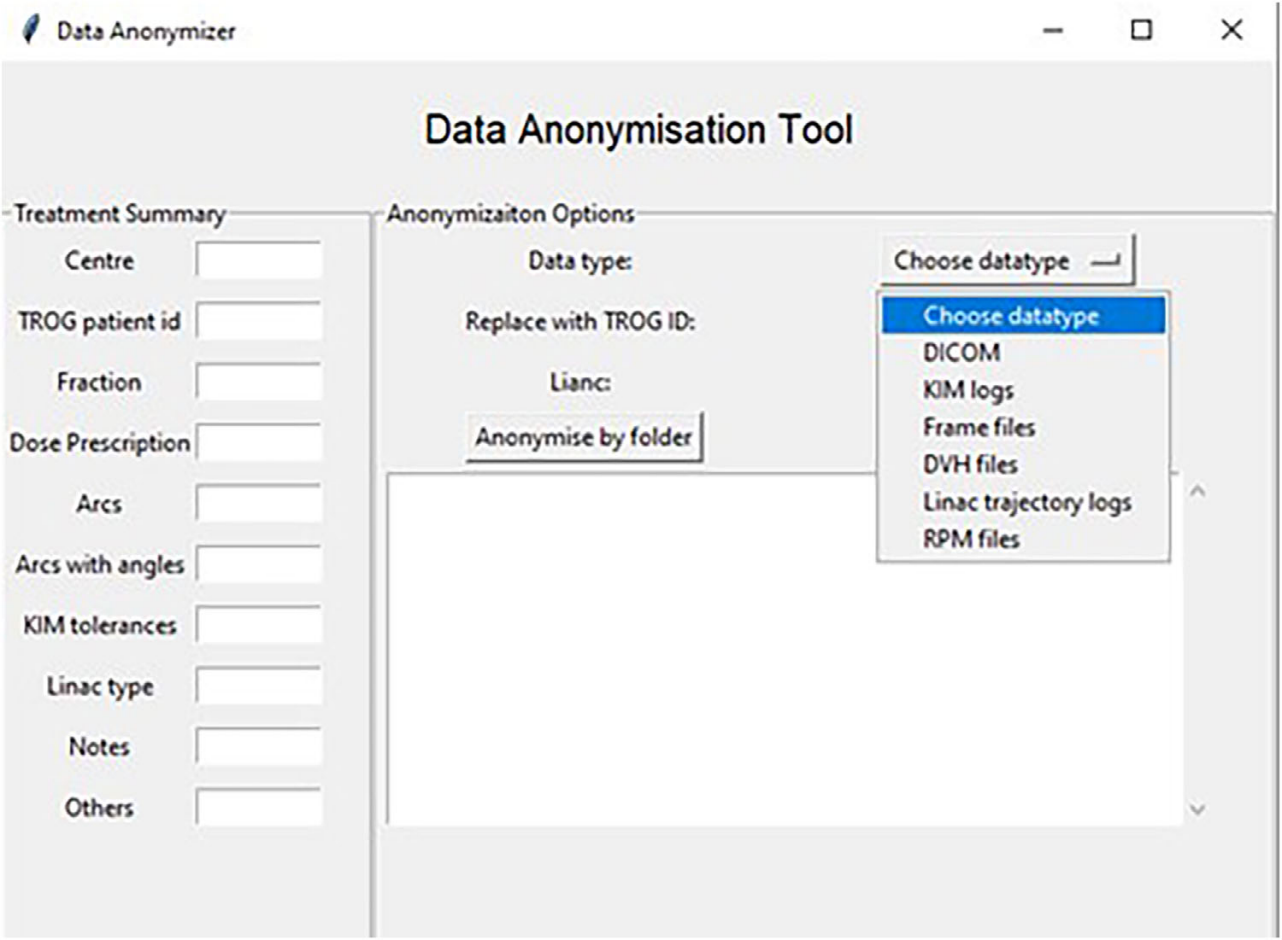
The challenges of data anonymization required the development of a dedicated tool. The data anonymizer is also open source.

One of the challenges of the SPARK dataset was data anonymization. This problem was solved by developing a dedicated tool. The interface is shown in Figure 3. The data anonymizer is also open source, found as part of the SPARK data analysis suite at https://github.com/Image‐X‐Institute/SPARK_Data_Analysis_Code/tree/main/Data%20anonymisation.

## 4 DISCUSSION

There are a number of potential applications of the data, with the primary application being the further development of real‐time IGRT methods to improve patient outcomes. These methods could include kilovoltage‐based IGRT approaches, and kilovoltage/megavoltage applications such as that developed with demonstrated clinical benefit at the Memorial Sloan Kettering Cancer Center.^12^ Multiple image segmentation, image processing, and noise reduction methods could be investigated. An obvious application is the development of artificial intelligence (AI)‐based applications. All AI and non‐AI algorithms are data‐hungry and benefit from large amounts of data for training, validation, and testing. The SPARK dataset contains approximately 1 million images.

The dataset will also be useful for development of motion‐included dose reconstruction methods and related work, as well as for investigating the patient treatment outcomes in this study with other types of motion adaptation methods. Tumor and normal tissue control modeling, and PSA kinetics could be investigated. Given the high treatment accuracy, the dataset is ripe for investigating the relationship of patient outcomes to vessel‐sparing radiation and functional anatomy‐based preservation for erectile function sparing.^13^

kilovoltage‐based real‐time IGRT applications necessarily add a dose to the patient. Many strategies could be used to reduce the amount of dose to the patient, by reducing the imaging frequency at the cost of reduced motion detection, or reducing the beam current at the cost of lower marker detection accuracy. The dataset could be used to evaluate the optimal tradeoff between patient dose and targeting accuracy.

**FIGURE 4:**
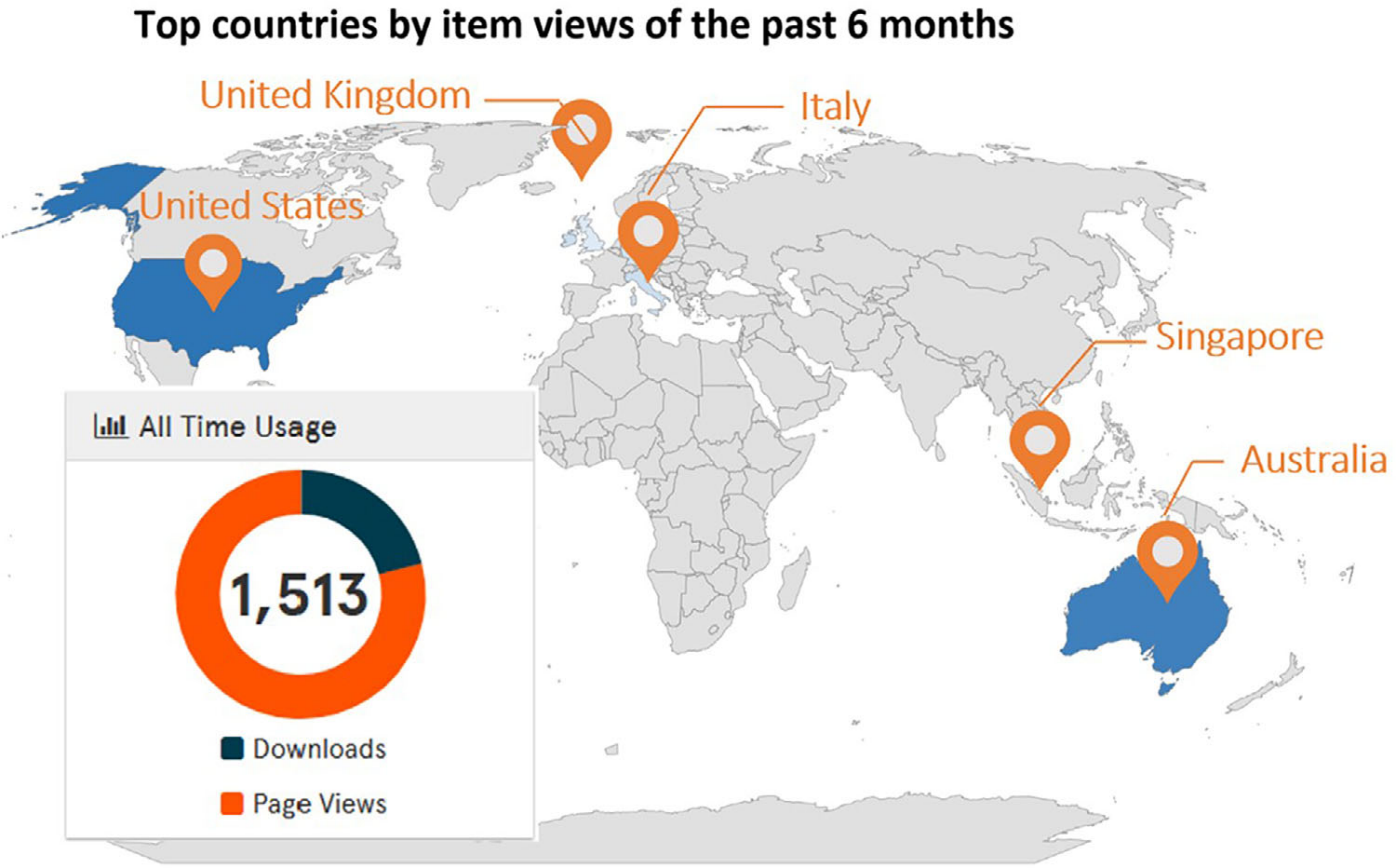
The SPARK user statistics measured from June 13, 2023 to April 24, 2024. The downloads value refers to the ReadMe file, which is the only download metric tracked.

Demonstrating the potential of the SPARK dataset applications, some of the user statistics for the ReadMe file download and page views are shown in Figure 4. Note that only the ReadMe file downloads are tracked.

## 5 CONCLUSIONS

Aligned with the US National Institutes of Health initiative,§ and the FAIR Principles,** the Trial Management Committee of the TROG 15.01 SPARK clinical trial supported the public sharing of the clinical trial data and accompanying analysis code. There are a number of potential applications of the data, with the primary application being the further development of real‐time IGRT methods to improve patient outcomes. The dataset has been used in eight publications to date and the ReadMe file has been downloaded over 300 times, indicating interest in the use of the data for further scientific research. Below is a list of publications that utilized the SPARK dataset:

1. Keall et al., Int J Rad Onc Biol Phys. 2020;107(3):530‐538.
2. Nguyen et al., Radiother Oncol. 2017;123:37‐42.
3. Keall et al., Radiother Oncol. 2018;127:6‐11.
4. Hewson et al., Med Phys. 2019;46(11):4725‐4737.
5. Wolf et al., Radiother Oncol. 2019;136:143‐147.
6. Hewson et al., Radiother Oncol. 2020;151:234‐241.
7. Mejnertsen et al., Phys Med Biol. 2021;66(6):065027.
8. Sengupta et al., Med Phys. 2023;50(1):20‐29.

## CONFLICT OF INTEREST STATEMENT

P. Keall is an inventor on a patent related to the KIM technology that is licensed to Varian Medical Systems by Stanford University. P. Keall, D.T. Nguyen, and R. O'Brien are inventors on additional patents/patent applications related to the KIM technology that has been assigned to the company SeeTreat. P. Keall and D.T. Nguyen are founders and directors of SeeTreat. J. Booth, N. Hardcastle, and R. O'Brien are employees of SeeTreat.

## Footnotes

†
https://www.ncbi.nlm.nih.gov/books/NBK285999/ accessed 2024‐01‐24.

‡
https://www.go‐fair.org/fair‐principles/ access 2024‐01‐24.

§
https://www.ncbi.nlm.nih.gov/books/NBK285999/ accessed 2024‐01‐24.

**
https://www.go‐fair.org/fair‐principles/ access 2024‐01‐24.
